# Early Classification of Intent for Maritime Domains Using Multinomial Hidden Markov Models

**DOI:** 10.3389/frai.2021.702153

**Published:** 2021-10-07

**Authors:** Logan Carlson, Dalton Navalta, Monica Nicolescu, Mircea Nicolescu, Gail Woodward

**Affiliations:** ^1^ Department of Computer Science and Engineering, University of Nevada Reno, Reno, NV, United States; ^2^ NASA Jet Propulsion Laboratory, Pasadena, CA, United StAtes

**Keywords:** machine learning, multinomial HMM, maritime, intent recognition, behavior recognition

## Abstract

The need for increased maritime security has prompted research focus on intent recognition solutions for the naval domain. We consider the problem of *early classification of the hostile behavior of agents in a dynamic maritime domain* and propose our solution using multinomial hidden Markov models (HMMs). Our contribution stems from *a novel encoding of observable symbols as the rate of change* (instead of static values) for parameters relevant to the task, which enables the early classification of hostile behaviors, well before the behavior has been finalized. We discuss our implementation of a one-versus-all intent classifier using multinomial HMMs and present the performance of our system for three types of hostile behaviors (ram, herd, block) and a benign behavior.

## 1 Introduction

The ability to understand the intention of others is of great importance for many application domains, either to enhance collaboration among members of a team, or to detect potential threats posed by enemy agents. Recently, there has been an increased interest in using artificial intelligence technologies for security and defense applications, in order to reduce the danger for the people involved. However, the current systems deployed by the US Army (e.g., Hermes) and the US Air Force (e.g., MDARS Shoop et al. (2006)) rely heavily on input from a human operator who assesses the situation and takes a decision. Given the threat of attacks on large ships performed by swarms of small boats, whose occurrence has increased in recent years, there is a great need of systems that can detect potentially threatening situations at sea. In addition, due to the numerous activities that the crew is required to perform on board the ship, and in areas of high boat traffic (such as harbors), there is a high chance that some threatening activities would go unnoticed. As a result, an automated system for understanding the intentions of surrounding boat traffic would be of high importance.

Intent recognition is a classification task with the goal of identifying what other agents aim to do, from their current actions up to the current moment of time. Intent recognition bears similarity to activity/goal recognition in that it uses time series data to classify the actions of an agent or agents. Unlike *activity recognition*, which seeks to identify actions that have already been completed, intent recognition has to address the challenge of identifying the intentions of agents *while their actions are still unfolding* and *before their goals have been completed*. In issues of security, this distinction is important as intent recognition allows sufficient time to react to the intentions of other agents. The information gathered from intent recognition can then be used in artificial intelligence applications to understand environments and plan future actions. In *goal recognition*, activities are modeled as sequences of actions that lead to goals. In contrast to intent recognition, this method allows for detecting a goal only after it has been achieved, which does not permit early detection of potentially threatening situations.

A second challenge that needs to be addressed in this context is the fact that the patterns for hostile behaviors do not have a fixed-length representation. Furthermore, transitions between such behaviors may happen at any time in a continuous stream of observations. To properly identify such behaviors in the ongoing stream of observations, the system must be able to quickly detect when such transitions occur, while at the same time it should identify the signature of the corresponding behavior. The solution we propose is to employ a sliding window of observations that is being used for behavior prediction, which allows the detection of transition from a behavior pattern to another.

Hidden Markov Models (HMMs) have typically been used for activity modeling and recognition. They consist of a set of states, with transitional probabilities between them. The states themselves are not directly observable and only a set of visible symbols can be detected. We propose to model the interactions between agents in the world using a novel formulation of Hidden Markov Models, adapted to suit our needs. The distinguishing feature in our HMMs is that they model not only transitions between discrete states, but also the way in which parameters relevant to an activity (e.g., relative positions, headings of two boats) change during its performance. This novel formulation of the HMM representation allows for recognition of the agents’ intent well before the underlying actions are finalized. In this work we focus on detecting three different hostile behaviors: ram, herd and block, as well as on the ability to infer a benign navigation pattern. The three hostile behaviors have been chosen due to their relevance to the naval domain, which we have acquired from subject matter experts in the Naval domain.

## 2 Materials and Methods

### 2.1 Related Work

The problem of understanding human activities has mostly been addressed from the perspective of activity or plan recognition, with Bayesian inference and probabilistic context free grammars as the most frequently used techniques. While these methods have proven successful in numerous applications, most often the focus is on recognizing activities after they have been performed. In addition, the complexity of the inference may become impractical for large problems and real-time applications.

In the realm of Bayesian inference, Charniak and Goldman Charniak and Goldman (1993) propose plan recognition Bayesian networks to encode relationships among events, and use standard Bayesian inference techniques to compute posterior probability distributions over potential plans. These methods rely on a probability distribution over a set of observed events. For practical real-world applications the number of observations can grow significantly, making inference impractical. Dynamic Bayesian Networks provide a more compact representation of the past observations, improving the efficiency of the inference process Neapolitan (2003). However, for many practical plan recognition domains, the size of the representations can lead to intractable inference. Methods for approximate inference can be used in particular domains Lesh and Allen (1999), but still do not achieve real-time performance.

Given the hierarchical structure of human activities, which is very similar to that of sentences in a natural language, probabilistic context free grammars (PCFGs) Manning and Scutze (2003) have been successfully used in interpreting activities in video sequences Brand (1997), Ivanov and Bobick (2000), Moore and Essa (2002), Minnen et al. (2003). However, PCFGs are limited in the types of queries they can answer and typically require that the entire observation sequence be available before inference can be performed. A solution to this problem has been proposed by Pynadath and Wellmann Pynadath and Wellman (1996), who provide a method for constructing a Bayesian network that represents the parse trees given by a PCFG. An application of this method Kitani et al. (2005) demonstrates the ability to recognize temporally overlapped activities by constructing a dynamic Bayesian network representation of a PCFG. The method has been applied off-line, on a set of existing videos, which is unsuitable for real-world applications, where inference should be performed in real-time and the observations only become available at run time. Pynadath and Wellman introduce probabilistic state-dependent grammars (PSDG) Pynadath and Wellman (2000) to incorporate an agent’s internal and external state as contextual information. The grammars exploit particular independence properties of the PSDG language for efficient answering of plan-recognition queries in applications of traffic monitoring and air combat.

Specific applications of Markov processes for intent recognition is present in Sartea and Farinelli (2018), where Markov chains are used for detecting intelligent agent behaviors. The application is focused on categorizing malware behaviors, but it utilizes Markov chains for early identification of specific behaviors. Hidden Markov models have also been used with success for American Sign Language recognition Vogler and Metaxas (1999), Starner and Pentland (1997), speech emotion recognition Schuller et al. (2003), skill learning Yang et al. (1994), bioinformatics Yoon (2009), human identification Cheng et al. (2008), and action recognition Afsar et al. (2015). Quintero et al. (2017) presents an application of hidden Markov models for intent recognition in road transportation and discusses the benefit of early recognition of pedestrian intentions. Hidden Markov models are used here to model distinct pedestrian behaviors in a 3D environment.

For maritime domains, existing research is limited thus far to mitigating maritime piracy. In Jakob et al. (2012), a multiagent system simulation is implemented to model the activities of pirate vessels. In this research, the authors focus on the set of countermeasures available rather than early classification of the intent behaviors of vessels. Additionally, the focus on piracy makes an assumption that hostile maritime behaviors are limited to smaller vessels.

In this work we propose an approach based on Hidden Markov Models that enables early recognition and handles continuous data streams for the detection of threatening behaviors on a maritime domain.

### 2.2 Naval Domain Background

In the case of maritime intent recognition between multiple agents, the behaviors being observed can be classified as hostile and non-hostile. In hostile behavior, one agent is exhibiting some aggressive behavior towards another. Here, our agents are considered to be large ships (own ship) and smaller boats (outside agent). The specific hostile behaviors considered include BLOCK, HERD, and RAM as exhibited by outside agents in the following scenarios:

• *BLOCK*: An outside agent seeks to block the own ship from a destination by intersecting its trajectory.
• *HERD*: An outside agent seeks to herd the own ship toward a desired destination by approaching and maintaining a short distance at a specific angle.
• *RAM*: An outside agent seeks to ram the own ship by approaching very quickly from an orthogonal direction.

In the discussed explanations of the three hostile behaviors, it is clear that much of the relevant information includes velocity, acceleration, and heading in order to gain an understanding of an agent’s trajectory and therefore their intent.

### 2.3 Hidden Markov Models

The HMM is a probabilistic model built on the classic Markov chain, specifically a discrete-time Markov chain. A Markov chain consists of a finite set of discrete states *s*_*i*_. At each step, time step the system can be in any of these states and can transition to another state with probability *P* (*s*_*j*_ (*t* + 1)|*s*_*i*_(*t*)) = *a*_*ij*_, with *a*_*ij*_ being the transition probability of being in state *s*_*j*_ at time *t* + 1, given that the system was in state *s*_*i*_ at time *t*. This model is often and easily abstracted as a directed graph where the set of vertices represent the model’s state-space and the edges are weighted according to the transition probabilities between each state.

In contrast to classic Markov chains, in which each the states correspond to observable events, in HMMs the state of the system at time *t* is not directly observable, thus called “hidden”. Instead, a set of visible variables (states) *v*_*i*_, which are a probabilistic function of the hidden states is available. For each state *s*_*j*_, we thus have a probability of observing a particular visible state *v*_*k*_, given by *P* (*v*_*k*_(*t*)|*s*_*j*_(*t*)) = *b*_*jk*_, with *b*_*jk*_ denoting the emission probability of an observable. The structure of the HMM, which includes the hidden states *s*_*i*_ and the set of visible states *v*_*i*_, is assumed to be given, together with a training data set of observations (corresponding to the visible states).

Gaussian distributions and Gaussian mixture models are commonly used to model emission probabilities for continuous variables, while multinomial distributions are used for discrete observations. An HMM’s transition and emission probabilities are estimated using the *Baum-Welch algorithm* Rabiner (1989). Two questions can then be answered about an unseen ordered sequence of observations. First, what is the most likely series of states that generated these observations under the given model? Second, and more useful for our application: how likely is the given model to have generated this new sequence of observations? The likelihood calculated by answering the latter question with the *Forward-Backward algorithm* Rabiner (1989) is used for our intent classification.

The main contribution of our approach consists in choosing a different method for constructing the model. This new HMM formulation models an agent’s interaction with the world through the way in which parameters relevant to the task are changing (e.g., increase, decrease, stay constant, or unknown). This is in contrast with the traditional approaches that solely model transitions between static states. With this representation, the visible states encode the changes in relevant task parameters. The observable symbols alphabet for our system consists of all possible combinations of changes that can occur on these parameters, as described in Section 2.6.

We used the *hmmlearn* Python module to train and classify intentions with our HMMs. *Hmmlearn* is an open source module that implements three types of HMMs with an API similar to *scikit-learn* HMMLEARN (2018). The models implemented in *hmmlearn* consist of the *Gaussian*, *Gaussian mixed-model*, and *multinomial* HMMs, where the names refer to the type of probabilistic distributions that can be used for the emission probabilities. Due to the nature and variety in our features, we used a multinomial HMM and developed the feature pipeline described below.

### 2.4 General Approach

The proposed approach for early detection of behaviors in the naval domain consists of a *training* and a *classification* stage.

The training stage consists of the following successive steps: 1) data collection (Section 2.5), which provides multiple samples for each of the four behaviors (3 hostile, 1 benign), 2) feature engineering (Section 2.6), which provides the sequence of observable symbols computed from the raw data, and 3) model training, which uses the *Baum-Welch algorithm* Rabiner (1989) in *hmmlearn* to estimate model parameters (transition and emission probabilities) for each of the four behaviors. For training, we initialized both transition and emission probabilities with uniform distributions.

For classification, a subset of the data samples reserved for validation are processed through the following steps: 1) features are extracted from raw data similar to training, 2) a sliding window of frames is selected for classification (Section 2.7) after filtering possible missing mover frames (Section 2.8), then 3) classification is performed as follows: for each frame for which a classification will be attempted, the sliding window of observations is used as input for all of the HMMs. Using the *Forward-Backward algorithm* Rabiner (1989) we return a probability that the emissions were generated by a mover with the associated intent. The classification returned by the system is the intent associated with the HMM that returned the highest probability.

The following sections describe these stages in more detail.

### 2.5 Data Collection

**FIGURE 1:**
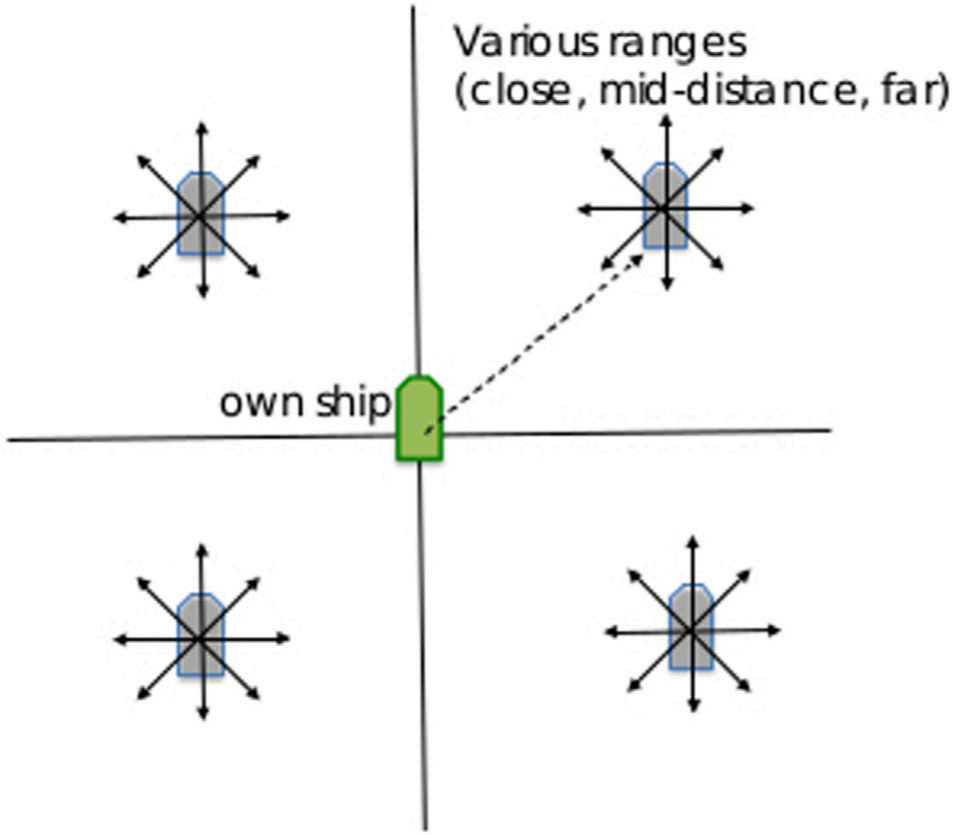
A depiction of the starting points of the potentially-hostile ship in each of the four quadrants.

The data used in this research was generated using simulation environments from the NASA Jet Propulsion Laboratory. The simulations considered the ocean as a two dimensional domain. The defending vessel began each scenario at the Cartesian coordinate (0, 0) and in each scenario it was tasked to reach a pre-specified goal. For each version of the scenario, the simulation began the potentially hostile vessel at unique coordinates in each of the four quadrants, and under the control of a specific controller for the four behaviors tested. A representation of the beginning locations of each vessel is shown in Figure 1. The uniqueness of scenarios at each starting position causes the hostile vessel to make slightly different movements in order to get into the correct position for the scenario’s hostile behavior, providing a comprehensive representation of each behavior.

It is important to note that during the scenario the defending vessel is using a controller for goal seeking, while following the International Regulations for Preventing Collisions at Sea (COLREGs), implemented as control rules in the simulator Kuwata et al. (2014). This means that the vessel may take evasive maneuvers in case the hostile boat interferes with its navigation. This has an impact both on the training and the testing phase. First, training data cannot be assumed to contain observations that pertain to a unique behavior, which affects the models. Second, testing data may contain scenario fragments that are not necessarily indicative of the main behavior, which impacts classification.

The controllers for the hostile vessel are implemented as state machines that allow transitions and maneuvers in response to the defending vessel, as follows. The HERD controller transitions between three states: 1) get in position (maneuver around the defending vessel to get to its side), 2) herd (slowly decrease distance between mover and target) and 3) match (mimic the target vessel’s behavior in speed/heading). The BLOCK controller transitions between: 1) get in position (maneuver around target vessel to get in front) and 2) block (decrease speed and distance in front of target vessel). The controller transitions back to get in position if the target vessel takes avoidance maneuvers. The RAM controller travels at maximum speed toward the target vessel, aiming to minimize the time and distance to a collision, while continuously adapting to the defending vessel’s avoidance maneuvers. For the BENIGN scenarios the vessel pursues a non-threatening, non-intersecting path with the defending vessel.

Given the scenarios conducted using the varying starting positions, the simulation then collected 1,000 frames of data for each scenario. In BLOCK and RAM behaviors, this number of data frames allows for the behavior to be executed multiple times. In HERD behaviors, this allows the behavior to be completely executed, including the early behavior as well as the final behavior where the hostile vessel has accomplished the goal and subsequently moves alongside the defending vessel for the remaining frames. Visualizations of each behavior is shown in the third column of Table 1. The information collected in each frame is detailed in Section 2.6.

**TABLE 1:** Graphs on the left represent the histogram of most emissions by behavior in the training set. Observable symbols are described in Table 2. Graphs in the center represent the prediction plots of a given scenario for each primary behavior. Graphs on the right represent the xy coordinate plots of the corresponding scenario for each primary behavior. The blue line represents the defending mover, wheres the red line represents the potentially hostile mover.

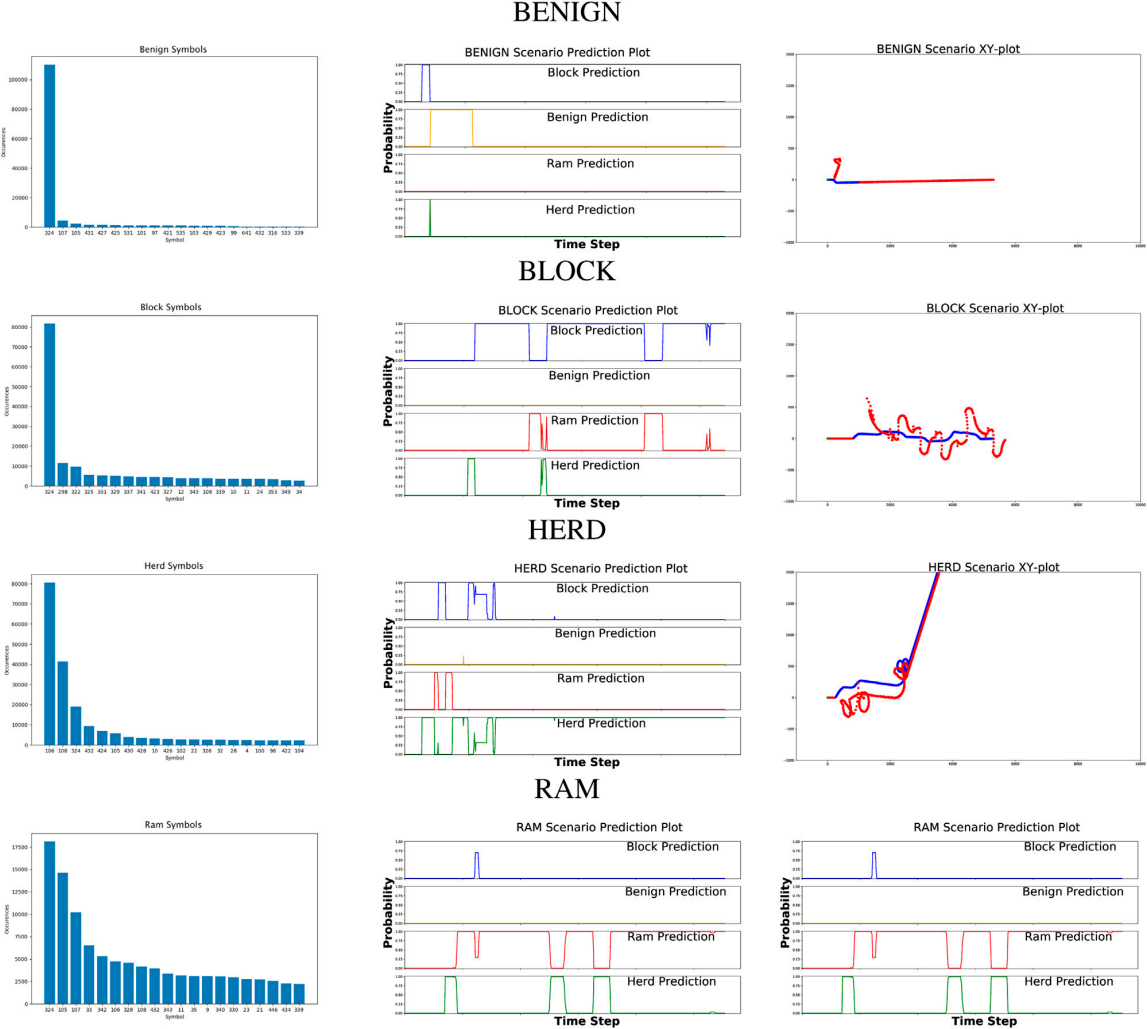

### 2.6 Feature Engineering

The features that our pipeline takes as input are continuous values. They consist of the following:

• Closest point of approach (CPA) distance and CPA time are the distance and time of travel that would minimize the distance between our vessel and the other vessel, assuming both vessels continue in a straight line and at a constant velocity.
• Cartesian coordinates of the defending and hostile vessels in a two-dimensional plane (*own_x*, *own_y*, and *other_x*, *other_y*, respectively).
• The velocity of each vessel in the direction of each axis (*own_vx*, *own_vy*, *other_vx*, *other_vy*).
• The heading of each vessel relative to the horizontal axis (*own_theta*, *other_theta*).
• The number of other vessels observed, *n*. All variables labelled *other* are retrieved for *n* vessels, including *CPA distance* and *CPA time.*

These features are received from a publisher-subscriber messaging queue along with additional information that we do not use, such as the length and width of the other vessel and the maximum speed of our vessel. The fields *own_vx*, *own_vy*, *other_vx* and *other_vy* from the current and previous frames are used to calculate the acceleration of all vessels in the direction of both axes, *own_ax*, *own_ay*, *other_ax*, and *other_ax*. The change in heading for each vessel, *own_dtheta* and *other_dtheta* are also calculated using the current and previous values for *theta*. If this is the first frame in which a vessel is observed, the vessel is assumed to have had an angle of 0 radians from the axis and $0\frac{m}{s}$ . At this stage, *CPA time* is set to a sentinel value for every vessel that has left our vessel’s view. Section 2.8 explains why this *missing-mover* distinction is useful. Finally, the combination of these features are used to create 7 discrete features which are codified as strings as specified in Table 2. These consist of:

• Relative angle: The other vessel is either facing toward or away from our vessel.
• Delta location: The distance between our vessel and the other vessel is either increasing, decreasing, or hasn’t changed beyond a threshold of 1 m.
• Delta speed: The velocity of the other vessel has either accelerated, decelerated, or has not changed by more than $1\frac{m}{s}$ .
• Delta angle: The other vessel is either facing more toward or more away from our vessel than it was last frame. This is constant if the angle has changed by less than 0.01 radians.
• Delta relative heading: Compared to the previous frame, each vessels heading may be more toward (decreasing) or more away (increasing) from each other. This is considered constant if the angle has changed by less than 0.001 radians. It is important to understand that the *heading* is distinct from the *angle* of a naval vessel; its heading is the direction of its movement, while its angle is the direction it is facing. These may differ to *sideslip*, or the tendency of ships to move sideways on the surface of the water.
• CPA time: The CPA Time is negative if the vessels are moving away from each other and positive if the vessels are moving toward each other.
• CPA distance threshold: The CPA distance of each other vessel is either above or below a threshold of 200 m.

**TABLE 2:** The seven features listed with their symbols and the meaning of each symbol.

Feature	Symbol: Meaning	Symbol: Meaning	Symbol: Meaning
Relative Angle	**00**: Facing Toward	**01**: Facing Away	
Delta Location	**10**: Moving closer	**11**: Moving farther	**12** Stationary
Delta Speed	**20**: Decelerating	**21**: Accelerating	**22**: Constant
Delta Angle	**30**: Turning toward	**31**: Turning away	**32**: Constant
Delta Relative Heading	**40**: Increasing	**41**: Decreasing	**42**: Constant
CPA Time	**60**: Positive	**61**: Negative	
CPA Distance Threshold	**70**: Above	**71**: Below	

Each individual HMM accepts only one emission per frame, so all of the discrete variables we have now computed must be encoded as a single integer per frame. For any frame, we concatenate the symbols in order to create a single string, called a *collapsed observation*. Then, we get the index of this frame’s collapsed observation from a sorted list of all possible collapsed observations. The index of our collapsed observation is what we consider as the emission of the hidden Markov model for a given frame.

### 2.7 Sliding Window Approach

Hidden Markov models are particularly useful in intent recognition because they process sequential data. In many intent recognition applications, the data being generated represents a behavior where the data is sequential (i.e. one frame after another). One of the underlying assumptions of the model is that only the state of the last timestep is relevant in determining the model’s current state. Accordingly, the transition probabilities are agnostic to any previous states other than the most immediate. However, the probability of a sequence of emissions of any length can be determined by simply multiplying the probabilities of each transition-emission combination in the sequence together, and summing these probabilities over the entire set of possible transitions.

This sequential benefit makes action recognition tasks straightforward Afsar et al. (2015) because the length of a sequence is known before it is analyzed. However, intent recognition requires that we identify an intended action before it is completed and before the time the action will take is known. Therefore, a *sliding window* of size *l*_*wdw* frames is used to make each inference. The last *l*_*wdw* frames are used in order to make an inference on the current frame, meaning that an inference cannot be made when less than *l*_*wdw* frames of the vessel in question have been observed. When a new frame is retrieved and its emission calculated, the oldest frame is deleted and a classification is made using the remaining *l*_*wdw*. The sliding window method has been used in various applications where the sequential data is used, including learning text-to-speech conversion Sejnowski and Rosenberg (1987), predicting protein structures Qian and Sejnowski (1988), and fraud detection Fawcett and Provost (1997).

**FIGURE 2:**
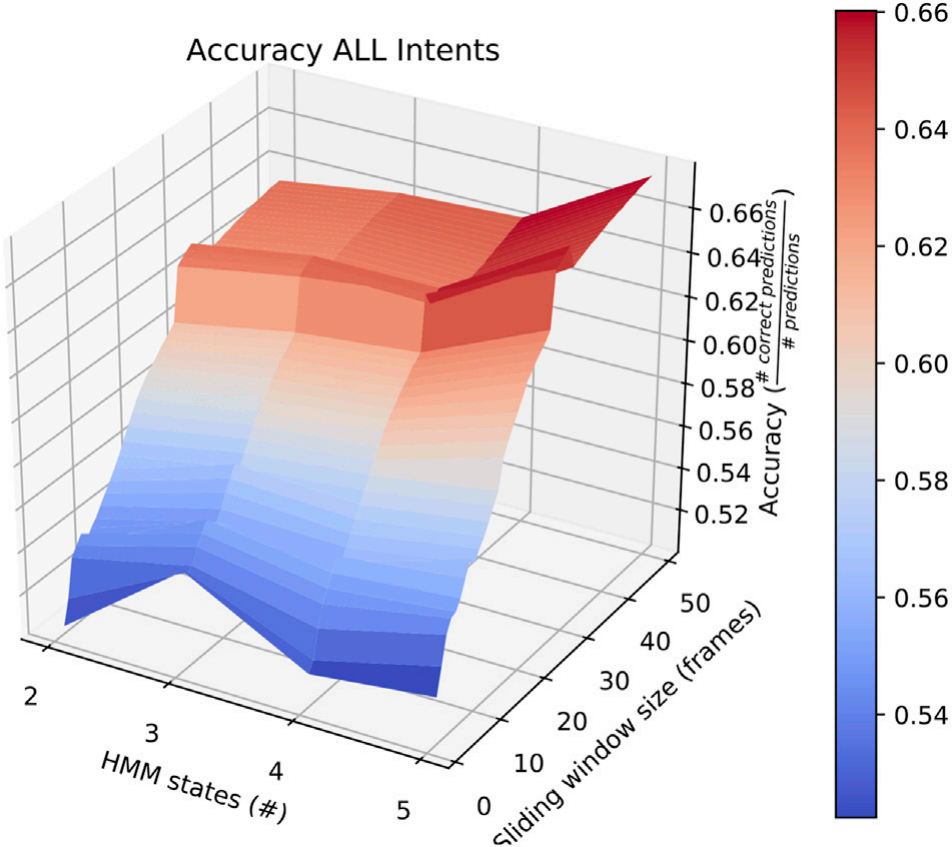
Surface plot showing the accuracy of the system given a set of options for sliding window length and number of states.

Sliding window lengths from 2 to 49 were tested along with varying number of hidden states, between 1 and 5. The average results of these tests are shown in Figure 2.

### 2.8 Missing-Mover Handling

At times, the other vessel may completely leave the range of our detection. Sometimes, the same vessel will then reenter view, and a new sliding window would have to be observed before another inference can be made. In these cases, the algorithm is unable to make classifications of potential hostile behavior, but must be able to make such classifications when the other vessel is again within range. This situation is managed using a threshold value that differentiates between erratic or temporary sensor information loss and the vessel leaving observation range completely.

**FIGURE 3:**
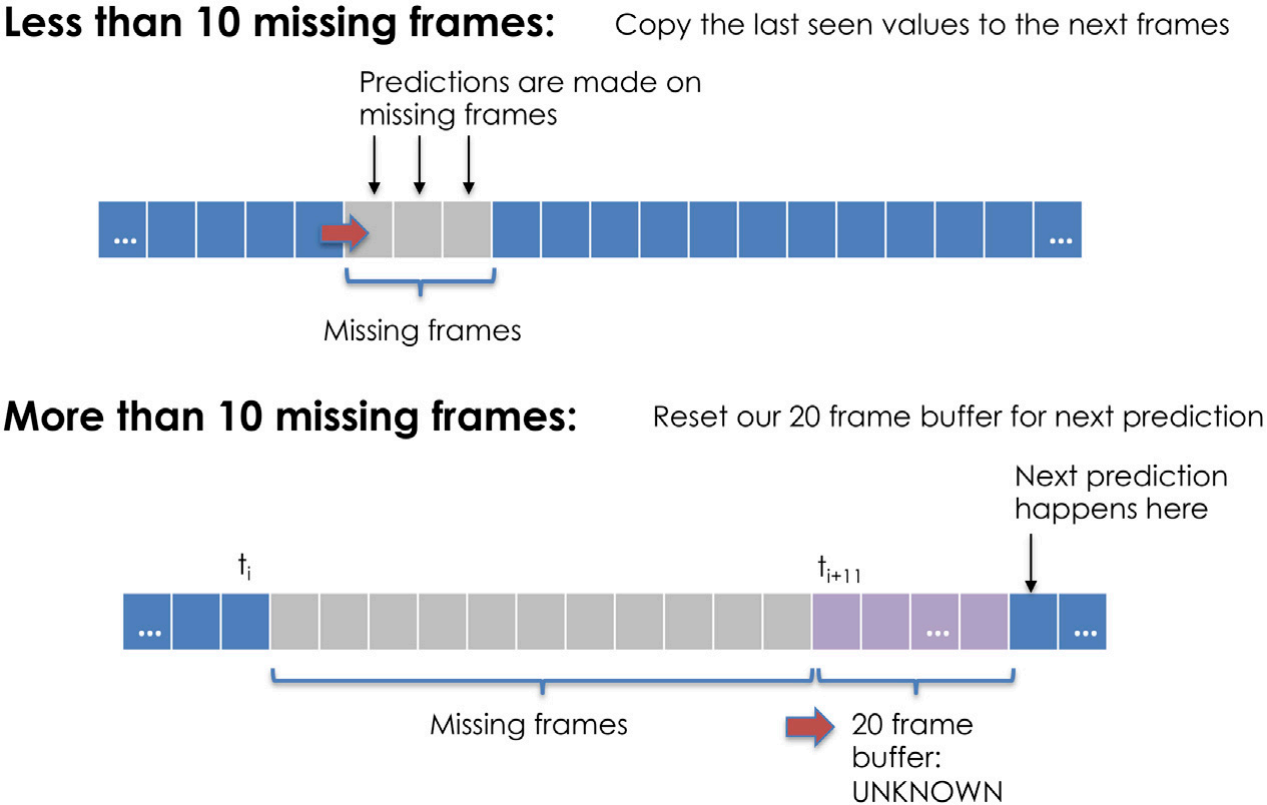
If the loss of sensor information is less than the specified threshold, the algorithm continues attempting classifications for the next observed data. If the number of missing frames exceeds the threshold, the algorithm must populate the rolling window before predicting vessel intent. This figure uses a 20 frame buffer window for display purposes, but the algorithm was trained using varying window sizes.

When a potentially hostile mover leaves range, the last observed emission is duplicated and added to the sliding window. Classifications are attempted using the resulting windows, but a counter keeps track of how many duplicates have been made. Once the counter exceeds a threshold of 10 frames, it may be possible that the mover has deviated from its last observed course, thus the entire window is deleted and the ship is assumed to be permanently out of range. This method is depicted in Figure 3.

## 3 Results

We aim to identify four specific behavior classifications of agents in maritime domains. These four behaviors are BENIGN, BLOCK, HERD, and RAM. We trained hidden Markov models using 1,104 simulated scenarios (276 of each behavior). These were used to test 8 scenarios representing each intent unseen scenarios using one-versus-all classification. Our classification system was evaluated based on the following performance metrics:

• Accuracy is defined as the number of frames with correct classification divided by the number of frames in which classification was attempted.
• Prediction Switches is the number of times that the prediction changed from frame-to-frame.
• Early Detection (Hostile) is the number of frames between the first frame in which the mover is detected and the first frame in which one of the hostile behaviors was predicted. This metric has a minimum of *l*_*wdw* − 1 and only applies to movers with hostile intent.
• Early Detection (Behavior) is the number of frames between the first frame in which the mover is detected and the first frame in which the correct classification is made. This metric has a minimum of *l*_*wdw* − 1.

First, the average overall accuracy of our classifier for all intentions and for each combination of number of HMM states and sliding window length was calculated. The most accurate consists of HMMs with 5 states looking at sliding windows 30 frames long. Classifiers trained and tested this way achieved an average accuracy of ∼ 67*%*, as depicted in Figure 2. We focus on this case when comparing performance on individual intentions below.

**FIGURE 4:**
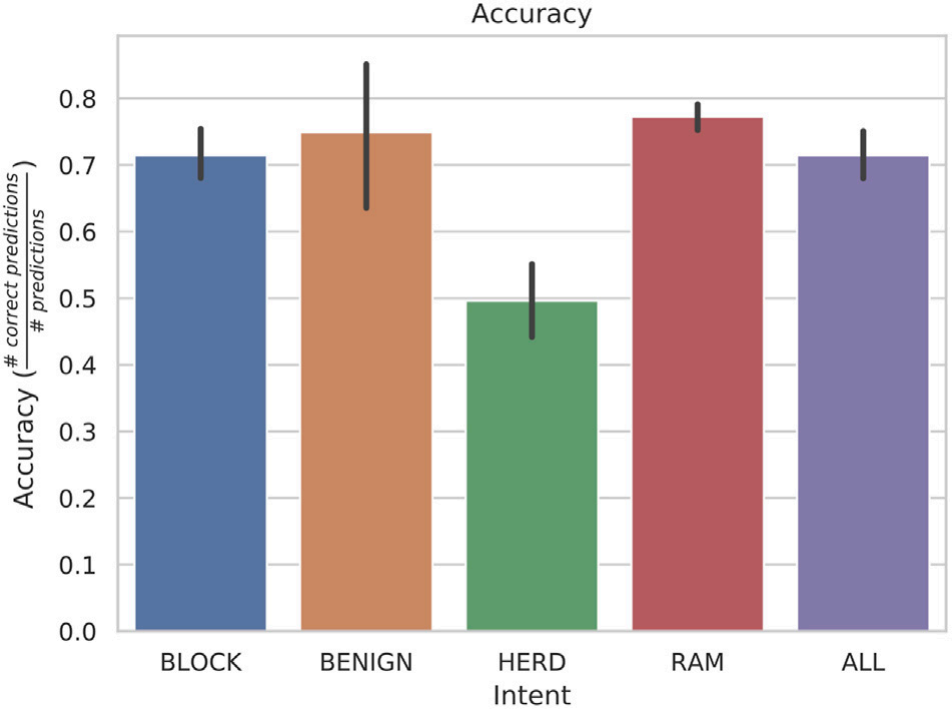
Accuracy averages with standard deviations from a rolling window of length 30 with 5 states. These were found to be the most accurate hyperparameters on average over all intentions.

Except for HERD behaviors, the accuracy of identifying each individual behavior is above 70*%*, as shown in Figure 4. We will discuss possible explanations for HERD’s performance after a short discussion of all of the listed performance metrics. While this figure does not sound impressive for accuracy, consider that the scenarios do not contain 100% the target behavior, due to the vessels obeying the COLREGs. Also consider that behaviors are similar in the early stages of each intent and so although hostile behavior is consistently predicted, misclassifications occur.

**FIGURE 5:**
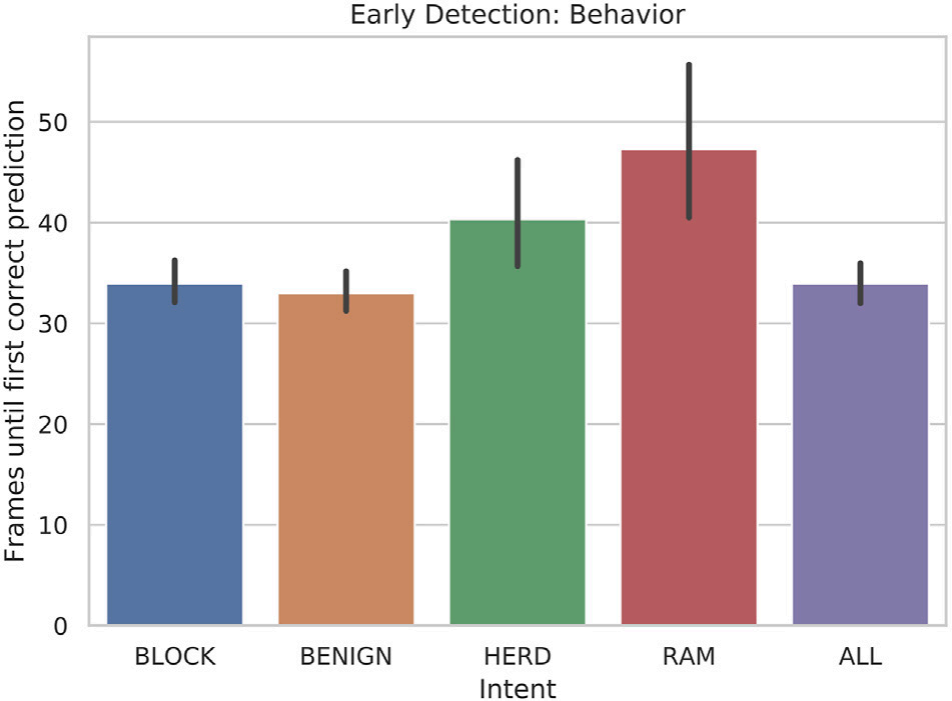
Bar plot of the early detection: behavior of scenarios when 5-state HMMs are tested with 30-frame sliding windows.

As seen in Figure 5, the classifier is able to consistently detect the primary behaviors less than fifty frames into the scenario, which has a total of 1,000 frames. The minimum possible early detection is 29 because *l*_*wdw* = 30. Considering this delay in the potential predictions, the algorithm is able to predict BLOCK, BENIGN, and HERD behaviors within 10 frames and RAM behaviors within 20 frames, on average. This is particularly important since each of the behaviors are time extended processes with significant overlap with respect to their observable symbols, as described below.

**FIGURE 6:**
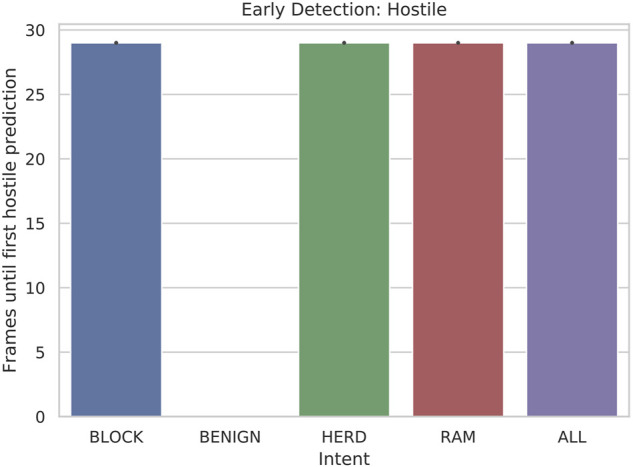
Bar plot of the early detection: hostile of hostile (RAM, HERD, BLOCK) scenarios when 5-state HMMs are tested with 30-frame sliding windows.

Our results for early detection (hostile) were very promising. We see in Figure 6 that hostile vessels are identified as hostile immediately when classification with a full sliding window becomes possible. This is important because, although an exact correct classification is not necessarily made, it is at least immediately obvious that defensive measures must be taken. Again, no plot is shown for BENIGN because this metric applies only when classifying hostile ships.

**FIGURE 7:**
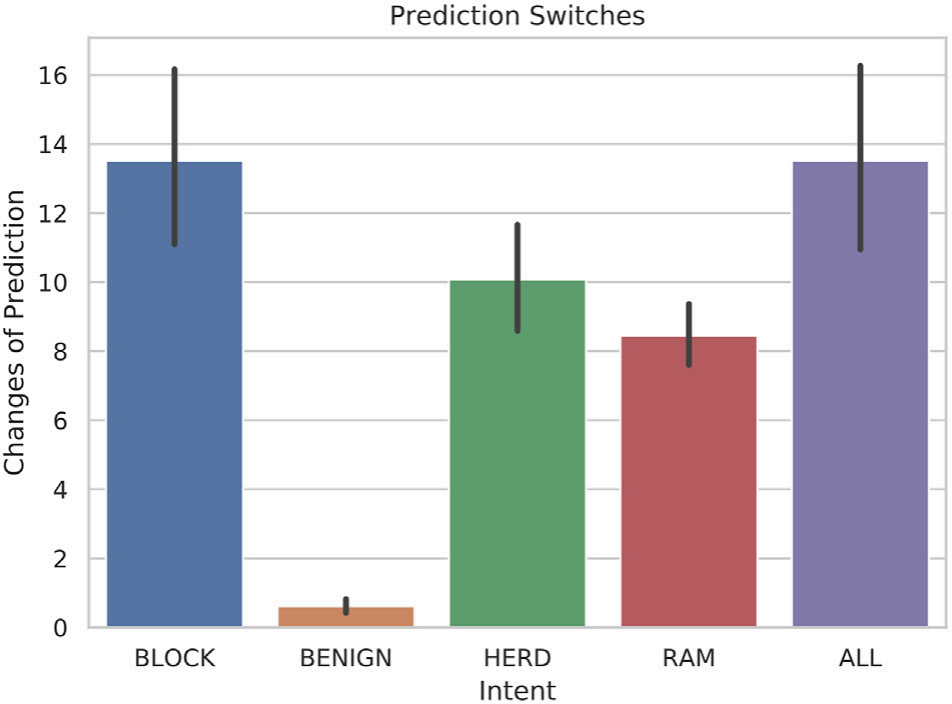
Bar-plots with error bars representing the quantity of prediction transitions between primary behaviors for each intent when 5-state HMMs are tested with 30-frame sliding windows.

Figure 7 illustrates the number of transitions between primary behaviors that the classifier detected on average for each intent. Due to the finite number of observable symbols and the similarities between behaviors, some oscillation is to be expected. The highest amount of prediction switches occurred with BLOCK scenarios, which is due to the repetition in the behavior throughout the scenario. The BLOCK scenarios have significant oscillation in order to exhibit the behavior, which can easily be confused with RAM behaviors, shown in the fourth row. The number of switches in predictions for BENIGN behaviors is minimal due to the simplicity of the scenarios, but also due to the incompleteness of data for BENIGN scenarios. The different cases of BENIGN behavior were scenarios without another vessel in range, a vessel that is only in range for a short time, a vessel that is mostly stationary, or a vessel that simply passes by without incident. In the hostile behaviors, however, the number of prediction switches is consistently below fifteen on average, and considering that the scenarios contained 1,000 frames, these results are encouraging.

In examining the symbol histograms on the left side of Table 1, we can see that the second and third most commonly observed symbol emissions in HERD scenarios were 108 and 324. These emissions map to collapsed observations “00112030406070” and “01102030406070” respectively. Both of these collapsed observations can be decoded using Table 2, but their meaning is not necessary to see the problems they cause when making predictions on HERD-intent vessels. Unfortunately, we can see in the histograms for the other three behaviors that 108 is also a common emission for RAM scenarios, and 324 is the most common emission of all scenarios combined. Still, these histograms were constructed using only the training data, so it is possible that some significant artificial difference exists between the training and test sets which would also explain the lack of testing accuracy in HERD scenarios.

## 4 Discussion

### 4.1 Future Work

Application of this work has been focused on simulation due to simplicity of training and testing the algorithms. In order to further verify the functionality of the trained HMMs and improve the training algorithm if appropriate, additional data collection would be necessary using increasingly sophisticated simulation techniques as well as on-water scenarios. On-water testing would be particularly useful not just for increased data collection, but to provide an industrial application of this work.

Moreover, since the data that has been collected and utilized in this research has been simulation-based, incorporating human navigation of Naval ships would provide realistic displays of hostile behavior in maritime domains and reasonable evasive maneuvers by the defending vessel. Even in the case of autonomous vehicles, on-water testing would probably produce different patterns due to unpredictable dispersal of waves and possible changes in wind and weather.

**FIGURE 8:**
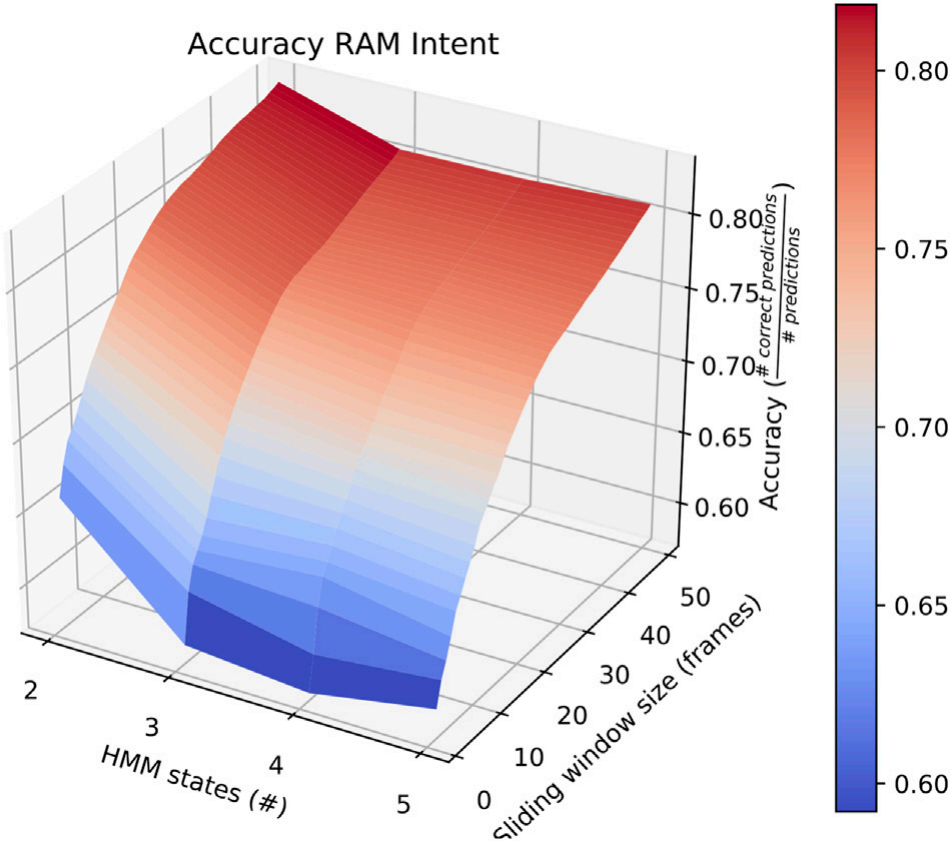
Surface plot showing the accuracy of the system on RAM scenarios given a large set of options for the length of the sliding window and the number of HMM states.

**FIGURE 9:**
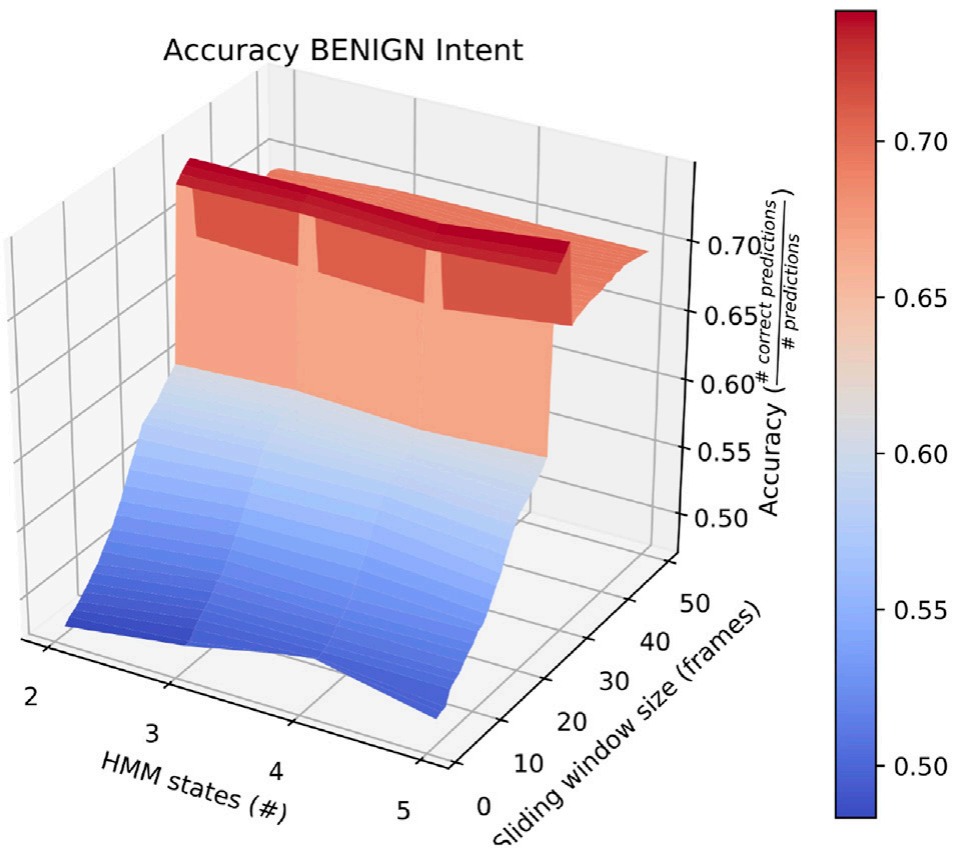
Surface plot showing the accuracy of the system on BENIGN scenarios given a large set of options for the length of the sliding window and the number of HMM states.

**FIGURE 10:**
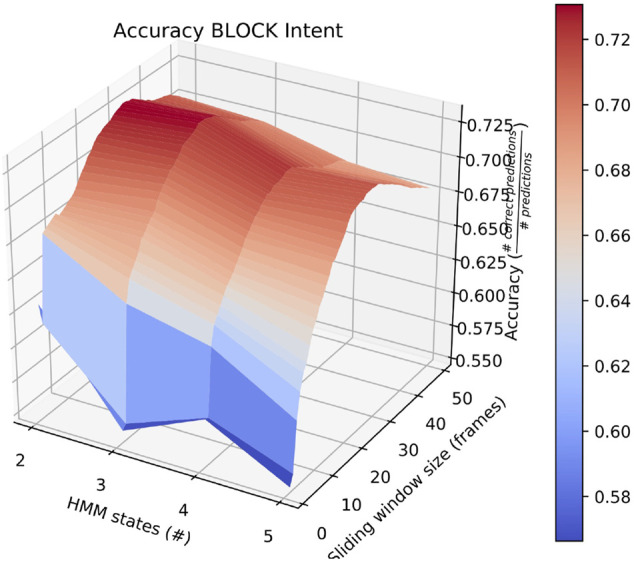
Surface plot showing the accuracy of the system on BLOCK scenarios given a large set of options for the length of the sliding window and the number of HMM states.

**FIGURE 11:**
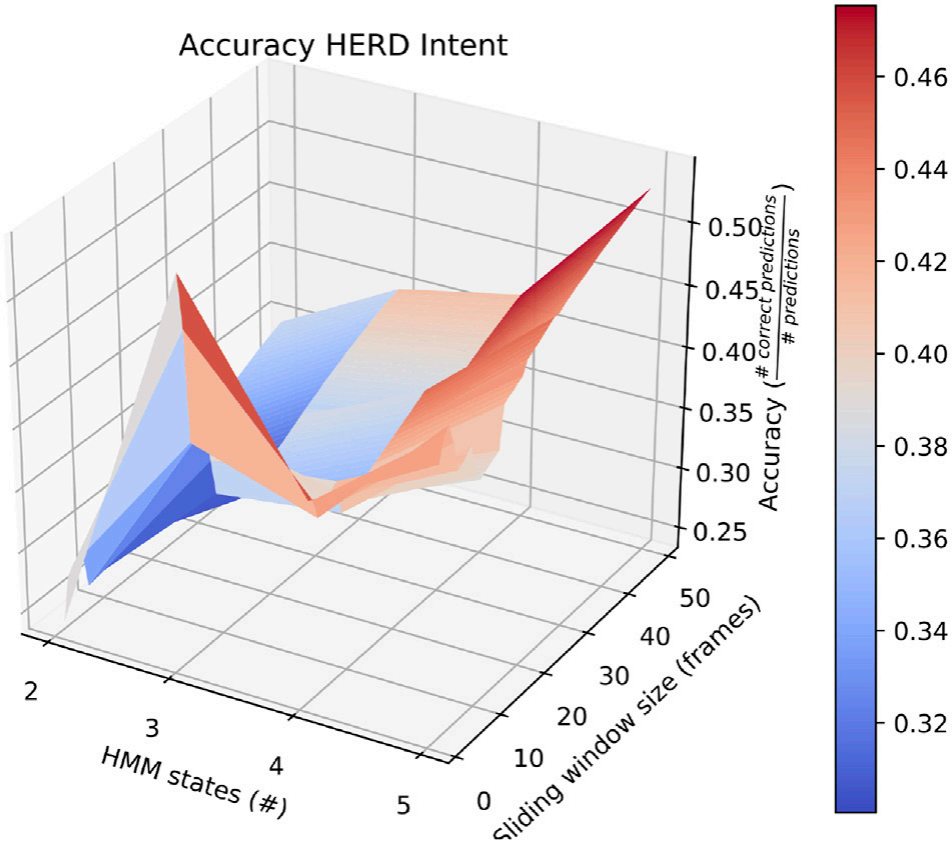
Surface plot showing the accuracy of the system on HERD scenarios given a large set of options for the length of the sliding window and the number of HMM states.

Also of interest is the fact that RAM scenarios achieve significantly better accuracy with a smaller number of states and with a longer sliding window. This is demonstrated by the surface plot in Figure 8. In fact, other than in HERD scenarios, the optimal selections for *l*_*wdw* and the number of HMM states are not usually the same as the ones we selected, and the highest average accuracy on a by-intent basis is always higher than the highest accuracy achieved on all intentions. Concretely, 74*%* accuracy is achieved on BENIGN scenarios when using 2-state HMMs with a 30-frame window (Figure 9), 73*%* accuracy is achieved on BLOCK scenarios when using 3-state HMMs with a 27-frame window (Figure 10), and 83*%* accuracy is achieved on RAM scenarios when using 2-state HMMs with a 49-frame window. Disappointingly, peak accuracy for HERD scenarios is only 54*%*, which is achieved when using 3-state HMMs with a 2-frame window (Figure 11). Given this information, it may be beneficial to test using a classifier where the number of states and length of sliding window are optimal for each individual behavior’s HMM. For this, model checking approaches that rely on pseudo-residuals Zucchini et al. (2016), cummulative distribution function (CDF) plots Altman (2004) or residual analysis and stochastic reconstruction methods Buckby et al. (2020) will be used.

Additional methods can be explored to improve the quality of the classification. First, we will investigate the use of alternative methods to expectation-maximization MacDonald (2014) in order to estimate the models’ parameters. Second, we will explore new models, such as Hidden semi-Markov models Guédon (2003) in order to account for possibly non-geometric sojourn distributions and provide a comparison of the results to our current work.

## 4.2 Conclusion

Automated solutions for intent recognition in maritime domains are limited despite successes in other domains. Hidden Markov models offer a solution to this disparity and we present a solution to the problem of *early detection and classification of hostile intentions* using multinomial HMMs. The method is based on a *novel encoding of observable symbols* as the rate of change for parameters relevant to the task. The results show that the trained models are capable of classifying multiple hostile intentions with very good accuracy. In addition, the models can detect these intentions during very early stages of the behaviors, giving ample time for the defending vessel to take evasive maneuvers. This approach can be applied to other domains that involve interactions between multiple agents, in order to facilitate their coordination and cooperation through implicit understanding of their intentions from observed actions.

## Data Availability

The datasets presented in this article are not readily available because the data is restricted by NASA JPL and Office of Naval Research protections. Requests to access the datasets should be directed to monica@unr.edu.
